# Cultural group selection and the design of REDD+: insights from Pemba

**DOI:** 10.1007/s11625-017-0489-2

**Published:** 2017-12-23

**Authors:** J. Andrews, M. Borgerhoff Mulder

**Affiliations:** 1 Department of Anthropology, UC Davis, Davis, USA; 2 Graduate Group in Ecology, UC Davis, Davis, USA; 3 Center for Population Biology, UC Davis, Davis, USA

**Keywords:** Cultural evolution, Multi-level selection, REDD+, Pemba

## Abstract

Evolutionary analyses of the ways humans manage natural resources have until recently focused on the costs and benefits of prudent resource use to the individual. In contrast, the fields of environmental resource management and sustainability focus on institutions whereby successful practices can be established and maintained, and the extent to which these fit specific environmental conditions. Furthermore, recent theoretical work explores how resource conservation practices and institutions can emerge through co-evolutionary processes if there are substantial group-level benefits. Here we examine the design of a prominent yet controversial institutional intervention for reducing deforestation and land degradation in the developing world (REDD+), and its ongoing implementation on Pemba Island (Zanzibar, Tanzania) to determine the extent to which the features of REDD+ might allow for the endogenous adoption of sustainable forest management institutions. Additionally, we consider factors that might impede such outcomes, such as leakage, elite capture, and marginal community participation. By focusing on prospective features of REDD+ design that could facilitate the spread of environmentally sustainable behavior within and between communities, we identify distinct dynamics whereby institutional practices might coevolve with resource conservation practices. These insights should contribute to the design of more effective forest management institution in the future.

## Introduction

Conservation is a cooperative social dilemma whereby individuals have to forego short-term benefits for future rewards (Hames 1987; Ruttan and Borgerhoff Mulder 1999; Smith and Wishnie 2000). In the case of forest-dependent tropical communities this often entails reducing the use of timber or other non-timber forest products. Accordingly, the quest for sustainable management of community-held natural resources is plagued by collective action problems. Currently, a major challenge lies in understanding how social systems move from sub-optimal environmentally harmful equilibria conferring immediate individual benefits towards Pareto-Superior, socially optimal, but costly cooperative solutions. Multiple disciplines address this dilemma. Evolutionary biologists explore potential mechanisms that encourage cooperation (Nowak 2006), resource economists’ socio-ecological systems research points to the central importance of institutions in long-term sustainable resource management (North 1990), and political scientists have developed a systematic framework of ‘best institutional practices’ for communities to manage local resources (Ostrom 1990). However, the processes under which these cooperative institutions evolve and spread are still opaque. This is a problem because as scientists we would like to make statements about how to make solutions attainable. Recently Waring et al. (2015, 2017) have proposed a cultural multilevel selection (cMLS) model to understand the development and transmission of cooperative, yet costly institutions for resource management. The argument hinges on the idea that cooperative conservation practices and institutions can evolve through ordinary cultural evolutionary processes (Boyd and Richerson 1985; Richerson and Boyd 2005). The central insight of this approach lies in recognizing that the relative importance of various scales of social life (such as groups, families and villages) varies in accordance with social and economic pressures (Andrews and Davidson 2013). The extent to which the key units of social life (i.e., villages) and natural resource management (i.e., conservation institutions) overlap creates conditions favorable to the emergence and possible spread of cooperative behavior (in a cMLS cascade, sensu Waring et al. 2015).

Here we explore to what extent a popular, yet controversial, instrument for mitigating climate change in the developing world—Reducing Emissions from Deforestation and Degradation (REDD+)—provides a framework within which the cMLS mechanisms can operate. The United Nations REDD+ program is a voluntary climate change mitigation approach designed to reduce global carbon emissions through forest protection (Angelsen 2008). Under community-based REDD+ programs, communities engage in self-policing to reduce deforestation and forest degradation in return for financial compensation through the sale of verified carbon emission credits on the voluntary international carbon market; in this sense REDD+ capitalizes on incentives that conservation scientists call performance payments, or payments for ecosystem services where rewards are proportional to conservation success (Ferraro and Kiss 2002; Wunder 2013). Despite controversy (Brown 2013) REDD+ has galvanized climate change action, garnering $6 billion in funding up to 2016 (Wolsosin et al. 2016). In this paper, we focus on the pending implementation of a REDD+ program in the community forests of Zanzibar (Tanzania), more specifically focusing on the island of Pemba, and explore its opportunities and constraints within the framework of cMLS.

Our argument is that REDD+’s design-principles create an ideal natural experiment, akin to a public goods/common-pool resource game that mimics the theoretical framework proposed by cMLS. When the net benefits to the community (arising from REDD+ performance payments) outstrip the cost to individuals of restraining forest harvest then individuals should preferentially adopt cultural institutions that aid costly forest conservation. Furthermore, if the REDD+ program effectively rewards communities in proportion to their relative performance in reducing deforestation, then imitation of local strategies, institutions and sustainability norms should spread to non-participating communities (Waring et al. 2015). We emphasize that the endogenous transmission and ‘scaling-up’ of REDD+ will be directly influenced by evolved social learning rules (e.g., payoff/conformist biased transmission) that affect individuals’ costs and benefits associated with adopting cultural traits. While these evolutionary biases are sculpted by differential reproductive success in environments of evolutionary adaptation, we make the key assumption that they remain relevant for influencing the impacts of economic incentives on the distribution and adoption of cultural traits in contemporary conservation dilemmas.

The purpose of this paper is to examine the design of REDD+ and to determine the extent to which the features of REDD+ might allow for a cultural multilevel selection cascade. Additionally, we consider the factors that might impede such outcomes, such as leakage, elite capture, and marginal community participation. Unlike Waring and Acheson’s (this volume) retrospective assessment of how the institutions of territoriality and harvest management practices might have arisen in the Maine lobster fishery, we focus on prospective features of REDD+ design that might facilitate the spread of environmentally sustainable behavior within and between communities across Pemba. We propose distinct dynamics whereby institutional practices might coevolve with resource conservation practices under REDD+. In this respect, we offer the beginnings of ethnography for how sustainable cooperative institutions emerge over time. We recognize that our research is in its initial stages as precise values of individual costs and group payoffs to forest protection are currently under collection.

The paper has seven sections. First, we introduce Pemba, focusing on its geography and history (2.1), contemporary economic livelihoods (2.2), and management of natural resources (2.3). There follows a brief primer on the REDD+ framework (3) and an overview of the theory framing cMLS (4). Our core argument lies in the following section on how cooperative institutions are maintained/supported (5) through both conformist biased transmission (5.1) and the use of institutionalized rewards and punishments (5.2), including a consideration of the effects of motivational crowding (5.2.1). We then assess how within the REDD+ framework cooperative traits might spread (6), both between individuals (6.1) and groups (6.2), ending with a consideration of how frontiers and leakage can foster the adoption of REDD+ (6.3). Our discussion considers the challenges and opportunities to REDD+ on the ground in Pemba (7). We reflect on the implications of a cMLS cascade of REDD+ institutions for the likelihood of payments emerging (7.1) and the impact of REDD+ on local power dynamics and inequality (7.2), before offering a brief conclusion (8).

## Pemba and its forests

### A brief geography and settlement history of the island

Pemba (1014 km^2^) is one of the two main islands of the Zanzibar archipelago, and lies 50 km off the coast of Tanzania. The island, geologically formed from the confluence of the Rufiji and Ruvu river deltas, supports high tropical forests and coral rag forests; its narrow inlets are home to over 6000 ha of mangrove forests.

Inhabited for over 20,000 years Pemba has long been known as the ‘Green Island.’ Its fertility, combined with its location, allowed it to play a central role in the development of the Swahili culture of the East African coast for well over a millennium, and become a major commercial clove producer, hence Zanzibar’s epithet as the “clove islands” (Sheriff 1987). Pemba rose to prominence in the late 17th century when it became part of the Omani Sultanate and (starting in the early 1800s) pivotal to global clove production (Goldman 1996), but reverted to being an agricultural hinterland when the larger southern island (Unguja) became the Omani capital in the 19th century (Sheriff 1987). In the late 19th and 20th centuries both islands came under British colonial rule. Currently, the islanders fall under the Revolutionary Government of Zanzibar as part of the United Republic of Tanzania.

### Economic and political life in the 21st century

With a population of 406,808, a density of 428 per km^2^ and a population growth rate of 3.1% (RGZ 2014) Pemba experiences high population pressure. The majority of Pembans live in rural communities that are typically highly dependent on forests and ocean resources, a balance that has oscillated over the last 1000 years of human occupation (Fleisher et al. 2015; Morales and Horton 2014). Villages are clustered into *shehia* (wards, comprising typically of 2000–6000 individuals). Of these rural dwellers 61% are classified as poor, or unable to meet their basic economic needs (RGZ 2014).

Principal livelihood occupations entail agroforestry (covering 44.1% of the island, RGZ 2014), with the production of rice, cassava, peanuts, coconuts, pineapples, cloves, mangos and other minor crop species in cleared areas and/or forest patches. Livestock raising, fishing (pelagic, reef and inshore), seaweed cultivation, small-scale marketing and (very limited) government employment provide additional food and/or income. Firewood collection, timber cutting, carpentry, harvesting of wild fruits, boat building, lime mining and coral quarrying are also very important activities, heavily impacting remaining areas of forest. In addition to these activities Pembans have historically depended heavily on clove production, an industry that has shaped much of the island’s ecology and society (see below), and there is a current rebound in the global price of cloves (Yussuf 2015). In short most Pemban communities can be described as forest-dependent (CARE 2015).

### Management of natural forests, forest conservation and initiating REDD+

Prior to the 20th century Zanzibar’s forests were community managed (Pakenham 1947; Shao 1992), yet during the 1964 revolution Tanzania nationalized all land creating a mix of open use and a few government managed forests. Escalating deforestation prompted the Finnish-funded Zanzibar Forestry Development Project (1980–1997), and a series of subsequent multilateral and non-governmental interventions for strengthening community forest management. These initiatives were formalized in the Forest Management and Conservation Act of 1996 (RGZ 1996) whereby the Department of Forests and Non-Renewable Natural Resources (DFNRNR) enter into Community Forest Management Agreements (CoFMAs) that empower communities to “plan, manage and benefit from local forest resources” (Benjaminsen 2014). This law endows communities with the right to harvest and sell forest products without paying royalties, to set and enforce bylaws, and exclude non-members. Under this law communities establish forest management groups known as *Shehia* Conservation Committees (SCC) elected from amongst members of the community.

As of 2017, the demographic and economic pressures that drive the need for new agroforestry land is the most immediate threat to Pemba’s remaining forests (CARE 2015). The second primary threat is fuel use, with 94.7% of all annual energy consumption coming from woody biomass firewood and charcoal (CARE 2015). The third main driver of deforestation is expanded clove production, capitalizing on new prices and grown with the agroforestry sector. Against this background, the REDD+ program was initiated across Zanzibar in 2010.

## A brief primer on REDD+ as a strategy

The REDD framework was first introduced to the world community at the 11th UN Framework Convention on Climate Change Conference of Parties (COP) meetings in Montreal in 2005. The motion initially proposed by Papua New Guinea and Costa Rica aimed to Reduce Emissions from Deforestation and Forest Degradation (REDD) and financially compensate countries for their efforts. The program was originally designed to facilitate and fund sustainable, low carbon growth in developing countries. In 2007, the first REDD framework was agreed upon at the 13th COP in Bali. By 2008 three additional targets—biodiversity conservation, sustainable forest management institutions, and enhancing carbon stocks—were added to meet the needs of diverse nations (Angelsen 2016). This more comprehensive program, called REDD+ , was promoted as a comprehensive framework for reducing carbon emissions through limiting deforestation and promoting carbon sequestration, and a “win–win–win” strategy for addressing climate change, poverty and biodiversity loss (Angelsen et al. 2012). Critical to its design is a reliance on market-based instruments (with institutional buffering by government agencies, Vatn 2010). Once communities have had their carbon stocks certified, they can sell these as credits on the carbon market (see “Discussion”).

**Fig. 1:**
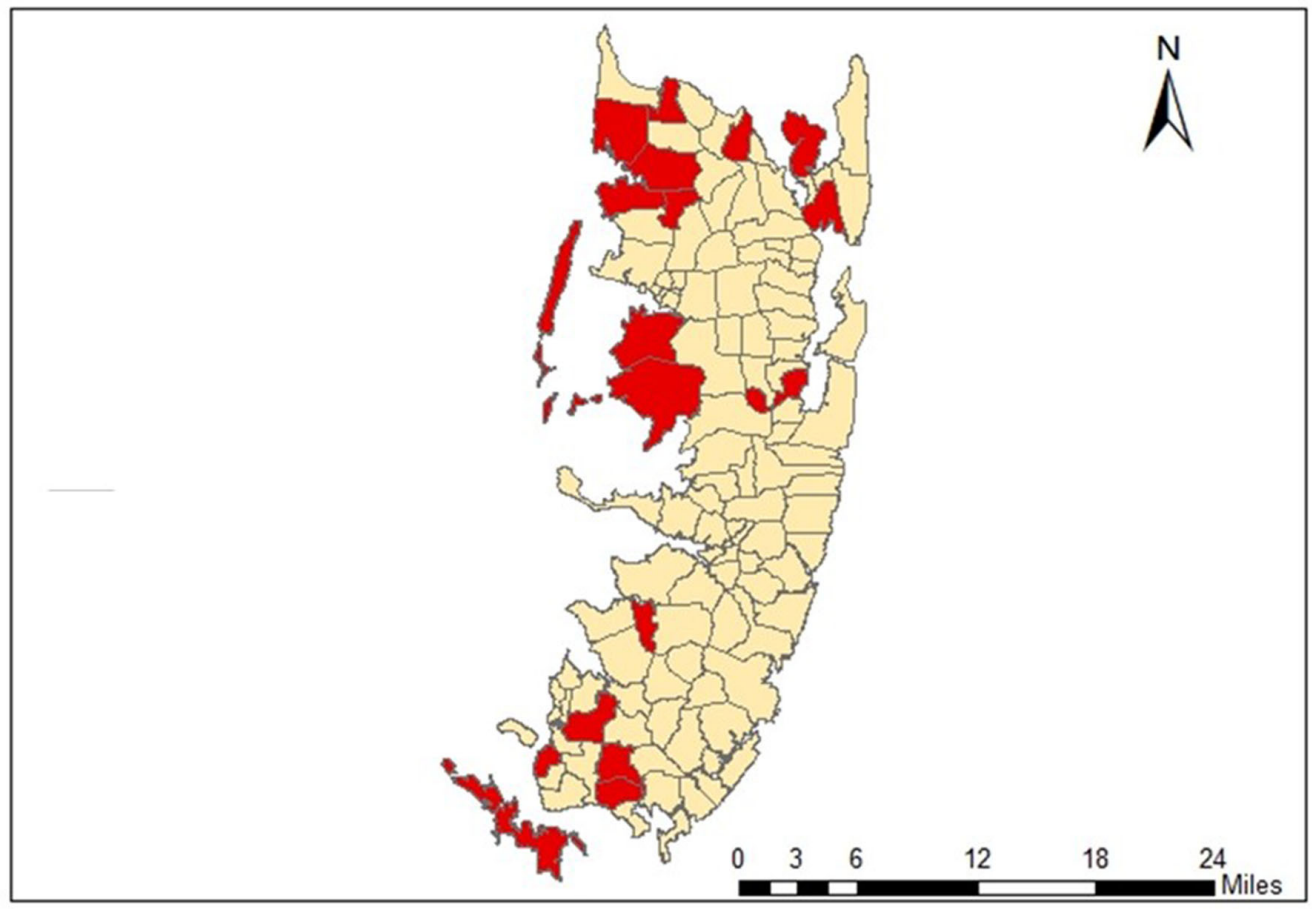
Map of Pemba showing the 18 *shehia* with Community Forest Management Agreements as of August 2015, hence termed REDD-ready. *Red* shading indicates presence of a CoFMA

In 2008 Tanzania was selected as one of several nations to pilot the REDD+ program under the financial support of (primarily) the Norwegian government (Angelsen et al. 2012) with eight projects (Robinson et al. 2013; Blomley et al. 2017) across distinct geographic areas, each managed by an international Non-Government Organization (NGO) for initial oversight and a local NGO to facilitate long-term implementation (Burgess et al. 2010). As such, one REDD+ project was initiated in 2010 under name Hifadhi ya Misitu ya Asili (HIMA, Protection of Natural Forests) in Zanzibar,

HIMA involved a four-way collaboration between CARE International, the forestry department, a San Francisco-based organization (Terra Global Capital) and a local facilitating umbrella NGO (see below); other organizations (not detailed here) assisted. The goal of the project, which lasted until 2014 (costing US$5.5 million with a no cost extension until August 2015) was to build effective (and strengthen existing) community forest management institutions across the islands on the basis of Free, Prior and Informed Consent (FPIC). The time line and activities are shown in Table 1, and the 18 REDD+ *shehia* on Pemba with formally certified CoFMAs are shown in Fig. 1.

**Table 1:** Time line of events relevant to implementation of REDD+ on Pemba

Date	Event
2007	REDD+ established (13th COP Bali)
2010	HIMA REDD+ pilot project established on Zanzibar
2010–2012	Baseline assessment and merging of previously established CoFMAs
2011	REDD+ readiness (training, capacity building, land tenure security, etc.) through HIMA
2011	HIMA Alternative livelihood interventions (including beekeeping, nursery development, efficient cooking stoves, tree-planting)
2012	JUMIJAZA established as umbrella organization
2012	HIMA Motivation/performance payments (first round, 18 *shehia* across Pemba)
2013	HIMA Motivation/performance payments (second round, 18 *shehia* across Pemba)
2013–2014	Woody Biomass Survey (DFNRNR)
2013–2014	Household SES Survey
2015	REDD-ready COFMA status certified for to 18 *shehia* across Pemba; CARE International and HIMA formally terminate; another 6 *shehia* complete REDD-readiness but do not receive formal certificates as not under Norwegian funding
2015	TGC submits project for Validation and Verification to certify carbon credits
2017	Stakeholders’ workshop

In 2012 HIMA established the NGO Jumuiya ya Uhifadhi wa Misitu ya Jamii Zanzibar (JUMIJAZA) to coordinate REDD+ over the islands, and to actively support communities in their conservation activities (see below). JUMIJAZA, supported by CARE International, played a major role implementing REDD-readiness programs, including distributing motivation payments according to the SCCs performance during the REDD-readiness period. It is also responsible for marketing carbon credits on the voluntary carbon market, and distributing the payments to the REDD-ready *shehia* (see below).

**Fig. 2:**
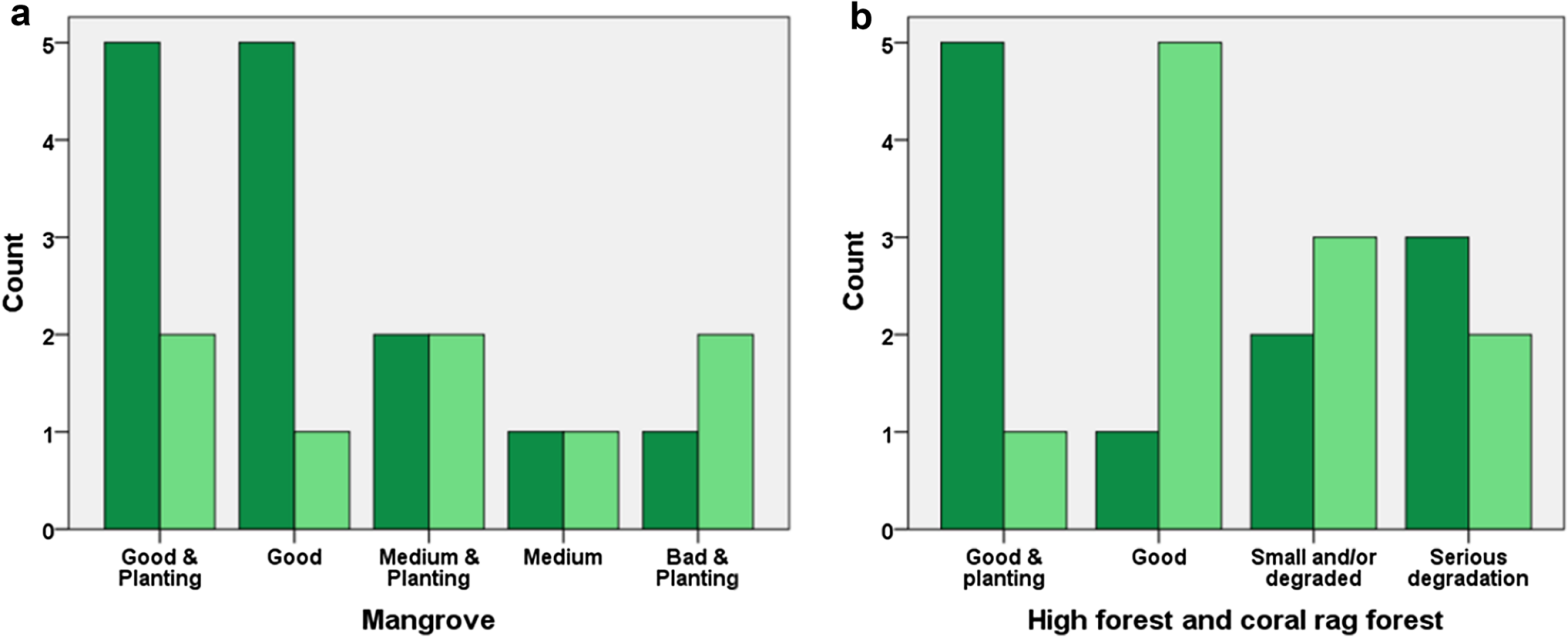
The state and planting of forests on Pemba at sites according to management status. *Shehia* with CoFMA (*n* = 11) *shaded dark green*; *shehia* without CoFMAs (*n* = 11) *shaded light green*. Sample limited to sites visited in 2015. **a** mangrove forests and **b** all other patches of high and coral rag forests

On Pemba 18 REDD-ready *shehia* with certified CoFMAs are overseen by JUMIJAZA; we use the terms REDD-ready *shehia* and *shehia* with CoFMAs interchangeably. These *shehia* elect an SCC of approximately 50 members from all the villages (ranging 4–12 villages) within the *shehia*, and make land use maps that designate High Protection Areas (HPA) within their jurisdiction. The purpose of the SCC is to provide awareness and education regarding the benefits of carbon sequestration to the community, monitor the forests, replant and conduct restoration where necessary, and administer alternative livelihood programs. It is important to note that the new SCC’s developed by REDD+ build on traditional community forest management of forests (Pakenham 1947), and indeed our anecdotal observations suggest considerable variability among *shehia* in the extent of their collective action prior to the HIMA program. Furthermore in the tradition of participatory forest management (Blomley and Ramadhani 2006) JUMIJAZA and the SCCs are assisted by the forestry department with policing (monitoring forests and fining illegal harvest) and with equipment for planting/restoration activities. Preliminary data based on expert (forestry department personnel) rankings suggest that the state and management of forests is best in *shehia* targeted by the HIMA project for REDD-readiness (Fig. 2, an association observed albeit only for mangrove forests). This is confirmed with data from interviews in randomly sampled households across 36 *shehia* visited in 2016 (Table 2); in the eyes of the community SCC in REDD-ready *shehia* evince improved management practices (planting and protection) when compared with the views of community members in *shehia* not targeted by HIMA. Interviews also reveal more effective communication of conservation objectives from REDD-ready SCC compared to those not participating in the REDD+ program. On the other hand there is no evidence of improvement in the control of leakage, nor any noticeable increase in arrests and fines in the REDD-ready *shehia*. Even more concerning though, and again in the eyes of the community, the SCC in REDD-ready *shehia* are more likely to be perceived as corrupt (Table 2).

**Table 2:** Forest management in REDD-ready and other *shehia* as reported by randomly sampled households within the *shehia*

	REDD-ready shehia^a^	Other shehia^a^	*t* Test and significance
Extent of leakage^b^	1.8 (12)	1.5 (16)	1.86 ns
Proportion of households in *shehia* stating that SCC give good explanation of conservation objectives to the community	0.26 (18)	0.09 (17)	3.38, *p* < 0.01
Proportion of households in *shehia* stating that SCC conduct arrests and propose fines for forest infractions	0.06 (18)	0.00 (17)	1.33, ns
Proportion of households in *shehia* stating that SCC conduct effective planting and restoration	0.39 (18)	0.26 (17)	4.70, *p* < 0.001
Proportion of households in *shehia* stating that SCC appear to be corrupt	0.15 (18)	0.04 (17)	2.37, *p* < 0.05

Responses came from open questions regarding the interviewee’s opinion of their *shehia* SCC, and were accordingly unprompted. Ns varied between 6 and 10 households per *shehia*

^a^N’s in parentheses denote number of *shehia*, and vary due to early modifications of the questionnaire

^b^Leakage ranks are calculated for each *shehia*, with high (3), average (2), low (1), and absent (0); as reported in interviews with randomly sampled households in each *shehia*, and averaged across households

Once Validation and Verification is complete (entailing, *inter alia*, the determination of a baseline historical rate of deforestation and a reliable methodology for measuring changes in land cover), carbon credits can legally be released for sale to JUMIJAZA. The carbon tonnage that is certified for sale is determined by comparing current rates of deforestation to the established historical rate to see, effectively, if carbon has been stored as a result of REDD+ when compared to the counterfactual (no REDD+). Thus, the more effectively Zanzibar sequesters carbon, the more carbon credits will be issued to JUMIJAZA. Using Landsat 8 satellite imaging, the individual success of particular *shehia* can be determined. As such *shehia* can be rewarded in proportion to their success in managing their forests. Such payments can *in theory* incentivize conservation if communities can also address relevant collective action challenges.

## Theoretical framework of cultural multilevel selection

In so far as conservation poses a collective action problem we turn to developments within evolutionary theory to understand the dynamics that lead to the evolution and spread of cooperative behavior. Historically, kinship, reciprocity, reputation, and spatial aggregations have all been invoked to explain the anomalously high levels of cooperation in humans (Nowak 2006). More recently, economists (North 1990), anthropologists (Boyd and Richerson 1985) and sustainability scholars (Ostrom 1990) note that the key missing ingredient in explaining human cooperation lies in cultural institutions and norms. Synthesizing theory from these diverse disciplines, Waring et al. (2015) present a cultural multilevel selection (cMLS) framework that offers theoretical basis of how sustainable cultural institutions evolve. Here we explore how REDD+’s incentive structure affects the adoption and spread of cooperative norms/institutions in light of cMLS.

The cMLS framework begins with the observation that most species including humans live in nested/structured populations. Individuals live in families, families are members of communities, and communities exist in broader structures. This nesting means that individual survival depends not simply on the action of an individual pursuing his/her own best interests, but is tied (at least in part) to the success of the group(s) to which they belong. Specifically, the degree to which individual survival co-varies with any other nested level provides a direct measure of the relative importance of that particular scale. Waring et al. (2015) argue that in resource-dependent communities, institutions will be preferentially adopted only when the collective action dilemma occurs at or below the level at which incentives are strongest. Specifically, if deforestation is the result of collective action failures at the *shehia* level, augmenting the covariance between individual success and *shehia* success should increase the probability of cooperative institutions emerging (or being adopted) through specialized social learning heuristics that will affect the rate and pattern of spread. To be clear, the spread of institutions through cMLS is not a process of direct natural selection, [like Darwin’s (1871) proposal that bravery would be selected through groups with brave warriors annihilating groups with timid warriors], nor is it a question of a neo-classical economic equilibrium. Rather it reflects the cultural dynamics that flow directly from the peculiarities of the evolved structures and biases that underlie human social learning (Boyd and Richerson 1985).

The central theoretical insight from cMLS theory is that the evolution of a trait depends directly on the relative strength of selection pressures at different hierarchical levels of social organization and the costs/benefits that trait confers across those levels (Okasha 2006). This basic axiom can be formalized in the Price equation below:

$$\bar{w}\Delta p = {\text{cov}}(P_{\text{g}} ,W_{\text{g}} ) + E{\text{cov}}(p_{{{\text{g}}i}} , w_{{{\text{g}}i}} ).$$

The equation measures the change in normalized trait frequency, $\bar{w}\Delta p$, as a function of the fitness contributions (or for our purposes economic payoffs/utility) from two distinct hierarchical levels. In its simplest interpretation, the equation partitions the total payoff of a trait into within-group interactions and between-group interactions. The second term on the right-hand side of the equation captures the individual level contributions to utility from a trait. This is measured by the expected covariance of a trait *p* in individual *i*, in-group *g*, and the associated payoff or fitness of that trait *w* to that particular individual. The first term on the right-hand side, measures the impact of the trait *P* on *group* g’s success/payoffs, *W*_g_. Crucially, the payoffs of a trait need not have the same sign not strength at the group and the individual level. This means that a cooperative act such as forest conservation can have a direct negative economic effect on payoffs at the individual level but a positive indirect effect at the group-level.

Multilevel selection requires two additional conditions beyond a population structured by nested groups. First, there must be heterogeneity between groups, such that groups vary in their outcomes. Between-group heterogeneity allows selective adoption of group-beneficial traits, preferential migration to more competitive groups, and the expansion of successful groups at the expense of less successful groups (Richerson et al. 2016) thereby increasing the frequency of traits associated with successful groups. Second, there must be high levels of homogeneity within groups. This homogeneity insures that group performance is highly correlated with underlying individual traits and high levels of homogeneity group reduce conflicts of interest and free-riding.

Turning to forestry, deforestation provides immediate economic benefits to individuals in the form of agricultural land, cooking fuel, timber products, and income from clove trees (around which small clearings must be maintained). Conservation, in contrast, is individually costly. These costs include opportunity costs from reduced harvesting and land clearance, effort towards replanting, time spent patrolling and monitoring forests and threats of retribution from issuing fines. While conservation provides group and individual level benefits through the provision of ecosystem services, the long-time horizon involved in forestry ensures that those benefits are subject to steep hyperbolic discounting. In the absence of supporting institutions selfish unsustainable behaviou, such as clearing land, harvesting firewood, ignoring bylaws and not punishing violations of forest use rules, is to be expected.

REDD+ projects change this equation by increasing the covariance between individual and group success. By design, REDD+ does this by issuing performance payments to *shehia* as a function of costly conservation behavior performed by individuals. These payments lessen (somewhat) the lag time between experiencing conservation costs and benefits, thereby reducing duration of discounting functions. Despite shifting the cost/benefit ratio in favor of conservation, REDD+ programs nevertheless still suffer from the threat of free-riders—individuals do better to continue to harvest the forest while at the same time reaping the benefits of REDD+ payments. However, as Waring et al. (2015) predict, it is precisely under these circumstances (if the payoffs to the group are large enough) that cooperative institutions (e.g., third party punishment) and norms should evolve that alter the payoff structure to make cooperation more attractive.

## The maintenance of cultural traits

Costly cooperative norms are maintained by a scaffolding of social learning rules and institutional arrangements (Henrich 2004; McElreath 2004). These transmission biases (Boyd and Richerson 1985) help individuals converge upon locally adaptive behaviors and often create high degrees of homogeneity within in-groups and variation between groups, thereby fashioning the necessary conditions for cMLS to operate.

Understanding the maintenance of costly conservation norms is central to deciphering how REDD+ projects can be sustained in the long run. The unpredictable returns from the voluntary carbon market means that the financial returns from REDD+ projects will periodically cross below economic and/or wellbeing thresholds identified in the Price equation. If payments are too low to cover opportunity costs, communities may reject (or drop out of) the scheme as is indeed happening in some places (Sunderlin et al. 2015). In the absence of strict economic incentives to motivate continued adherence to conservation norms, the role of non-economic forces for maintaining conservation norms becomes pivotal. In this section, we address three principal mechanisms that help drive the maintenance of cultural norms within groups: (i) conformist biased transmission, (ii) the mechanisms of rewards and punishment, and (iii) the potential for the crowding in/out of intrinsic motivations.

### Conformist biased transmission

Conformity, although often viewed as a hindrance to progress, is fundamental to the psychological architecture that underlies social learning in humans (Boyd and Richerson 1985). Conformist transmission is a cognitive learning bias that allows culturally naïve individuals to acquire adaptive cultural information at a relatively low personal cost. It does this by biasing individuals towards preferential adoption of the most common traits within a group, thereby ensuring a strategy that yields at least average payoffs.

As a learning strategy that is more sensitive to frequency distributions than payoffs, conformity can stabilize costly strategies even when shocks cause these strategies to lose strict payoff dominance. Additionally, insofar as conformist transmission promotes within group-homogeneity and between group heterogeneity, it also ensures that the necessary conditions for cultural group selection are met (Henrich 2004). By strengthening group identity, conformity increases the salience of group boundaries that are vital to sustainable management of common-pool resources (Ostrom 1990; Richerson and Boyd 2001). In short, conformist transmission can encourage the long-term stability of conservation norms in the face of fluctuating carbon markets.

Yet, for conformist biased transmission to operate effectively, the trait in question must be common in a population. Thus, conformist transmission will likely pay a weak role in the early spread and maintenance of cooperative conservation norms in the initial phases of REDD+ initiatives. Therefore, we must look to other mechanisms to account for the stabilization of costly traits, at least within the earliest stages when these traits are rare.

### Rewards, punishments and institutions

The most effective way of stabilizing costly cooperative norms is to modify the underlying payoff structure of the games people play in their social and economic interactions. This is done by altering incentives through provisioning benefits and/or administering costs. Typically, humans adjust these payoff matrixes through institutions. Institutions introduce new rules that govern how individuals interact in collective action dilemmas (Axelrod and Keohane 1985). By providing consistent and well-defined rewards and punishments for norm compliance, institutions modify an individual’s cost/benefit calculations thereby reducing both free-riding and time discounting that can jeopardize conservation efforts (Fehr and Gächter 2002).

The REDD+ framework addresses these features of human nature. First, economic performance payments operate to offset time discounting and compensate lost opportunity costs. Second, REDD+ institutions (such as REDD-ready *shehia* on Pemba) have the formal legal authority to monitor forests and administer punishments in the form of fines for violations of bylaws created by their CoFMAs. In this section, we briefly discuss the role of incentives (payments and punishments), leaving a full consideration of actual benefit sharing mechanisms (BSM) to the discussion.

With performance-based payments to the *shehia* being conditional on the degree of conservation success, the incentives for forest conservation shift from the individual to the group. Whether or not performance payments alter the underlying economic incentives sufficiently to change the social dilemma from a classic collective action problem to a simple coordination challenge depends on the utility gains to individuals; in the case of REDD+ these gains are determined by community payoffs that depend on an unpredictable international carbon market. In addition, the impact of these incentives will depend on how the benefits are distributed (BSM), whether the payments are issued directly to individuals, to the collective, or some combination of both (Agrawal et al. 2015; Kaczan et al. 2016). If they are issued to the collective, exactly what kinds of public goods are provided can directly affect the public goods production factor, allowing for small payments to have disproportionately large effects on utility. This means that understanding the underlying individual preferences for public goods is crucial for determining the utility derived from payment structures (Kaczan et al. 2016).

With respect to punishment, experimental research demonstrates its success in eliciting cooperation in collective action dilemmas; indeed humans show strong internal motivations to punish free-riding (Fehr and Gächter 2002). Furthermore, the willingness of individuals to punish is a significant predictor of successful community forest management in the developing world (Kosfeld and Rustagi 2015; Rustagi et al. 2010). Institutionalizing punishment to stabilize cooperative norms nevertheless raises complications. Social punishment is costly because it takes time and effort to monitor others’ behavior (Carpenter 2007), and those who punish frequently expose themselves to the threat of retaliation (Janssen and Bushman 2008); individuals therefore have immediate incentives to defect on the responsibility to punish norm violators, and to free ride on the altruistic punishment of others (Perc 2012). Adding subsequent layers of hierarchical organization, in this case the SCC, the collective action problem merely shifts the problem to a higher level of social organization.

Many solutions have been proposed to second-order cooperation problems, such as conformist transmission (Henrich and Boyd 2001), but in large groups the costs of diffuse monitoring become too large to sustain punishment, especially when there are significant benefits to rule-breaking. In light of this challenge, theoretical models (O’Gorman et al. 2009) and empirical evidence (Baldassarri and Grossman 2011; Traulsen et al. 2012) have shown that, in large groups, humans tend to solve the ‘second-order’ problem through ‘pooling punishment.’ Pooled punishment limits the right to punish to a small subset of the population, who are then compensated for their efforts (Sigmund et al. 2010; Traulsen et al. 2012). By restricting the right to punish, pooled punishment effectively creates institutional organizations that have an exclusive prerogative to use sanctioned force and thus can drastically modify payoffs and politics. This institutional formula is used by HIMA, insofar as SCCs have the right to arrest and propose fines on individuals breaking locally agreed on conservation practices. Nevertheless, pooled punishment only relegates the second-order punishment problem to a higher level—members of the SCC are still privately motivated to use their power over punishment to conduct illegal forest harvests, thus highlighting the need for effective leadership and supervision by the umbrella NGO.

#### Crowding out

Rewards and punishments do not always interact linearly or additively with pre-existing psychological motivations for cooperation. Motivational crowding occurs when an extrinsic incentive that was meant to increase cooperation (financial reward or punishments) reduces or has a diminished impact on the otherwise endogenous motivation to cooperate (Bowles and Polania-Reyes 2012; Frey 1997; Frey and Jegen 2001). By issuing rewards and punishments, REDD + projects can unintentionally reduce (or more rarely increase) pre-existing conservation motives of community members, which can be attributed to (i) putting a price on conservation effort, (ii) signaling bad faith in villagers’ intentions, and (iii) reducing individual autonomy (Bowles and Polania-Reyes 2012).

Motivational crowding raises an overlooked issue with cMLS. When cost–benefit calculations are used to determine the strength of selection, researchers often neglect non-material psycho-emotional benefits, such as the ‘warm-glow’ or reputational benefits that individuals derive from being altruistic and complying with social norms. This often means that the covariance between short-term selfish behavior and individual success is more complex than strict calculations based only on tangible direct material rewards. Our field observations of Pemban individuals, in multiple instances, contributing effort and cash voluntarily to faltering environmental NGOs with which they are associated attests to this point. Additionally, when incentives are introduced through contingency payments, the monetary rewards may actually decrease conservation efforts or at least have steeply diminishing returns on individuals’ motivation. The result would be that REDD + programs could actually have the opposite effect on forest conservation. However, this problem can be abated by developing incentive schemes and BSM that crowd in, rather than out, pre-existing motivation for conservation by tapping into existing individual preference structures (e.g., Kaczan et al. 2016).

## The spread of traits

The process of how cultural traits are spread across populations and groups is distinct from how they are stabilized. It is well acknowledged that the S shaped diffusion of innovations is at least partially accounted for by the cost–benefit calculations of individuals, particularly in social dilemmas (Greenhalgh et al. 2004) and network dynamics (Rogers 2010). Understanding the spread of traits with significant trade-offs at different levels of hierarchical organization has been a central focus of evolutionary theory (Smith and Szathmary 1997). Insights from cultural evolution stress how cultural transmission is necessary for multilevel selection to operate in humans (Boyd and Richerson 1985; Boyd et al. 2011). Here, using elements of a cMLS framework, we will address how payoff-biased transmission and external pressure generated from forest management practices within REDD-ready *shehia* can affect the endogenous spread of REDD+ institutions.

### Payoff-biased transmission among individuals

The core social learning mechanism that drives the spread of adaptive cultural traits is payoff-biased imitation. In the pursuit of high quality information, a culturally naïve individual can increase her probability of acquiring above average cultural traits by selectively imitating strategies with the highest payoffs (Baldini 2012; Boyd and Richerson 1985). Selection operating on this principle has endowed humans with an evolved psychological predisposition to preferentially attend to and copy successful and prestigious individuals (Henrich and Gil-White 2001). This trait allows for the rapid spread of adaptive behavior within populations, and can greatly increase the rate that populations move towards socially optimal equilibria, especially under rapidly changing environments (Kendal et al. 2009).

For payoff-biased imitation to operate, individuals must be able to identify and adopt strategies that have a strong positive covariance with evolutionary success. Outwardly visible displays of success such as prestige, wealth, and conspicuous consumption, are easily observable markers for success but they have a simple problem—they provide no information as to which behavioral traits led to such high payoffs (Henrich and Gil-White 2001). To increase the efficiency of payoff-biased transmission, the covariance among signals, payoffs and strategies must be clearly demonstrable. Highly visible public goods, such as dispensaries, schools and madrassa, built explicitly by REDD+ funding taps the underlying cognitive architecture of social learning and creates an easily identifiable link between the behavior of conservation protagonists and economic payoffs.

Note, group dynamics influence the effectiveness of payoff-biased transmission. Payments made at the group-level (*shehia*) do not affect variation in *intra*-*group* individual level payoffs, thus there is little chance for localized payoff-biased social learning. However, REDD-ready *shehia* can also use fines and social incentives to create covariance between individual forest harvesting practices and in-group individual payoffs. Additionally, if prestigious community members are elected to the SCC prestige- biased transmission can help spread conservation norms.

Therefore, when payments are issued to groups, payoff-biased transmission must primarily rely on inter-group transmission. This raises a problem insofar as norms are not easily transmissible between groups; this is because norms are often adapted to specific social and ecological contexts (Panchanathan et al. 2010), and because frequency dependent bias renders the novel traits of outsiders unacceptable (Boyd and Richerson 1985). Accordingly, over evolutionary time the costs of copying outsiders have sculpted the in-group/out-group psychology that places high psychic costs on adopting out-group behavior (Henrich and Boyd 1998). This out-group avoidance acts as major hindrance to scaling-up development interventions.

Recognizing these challenges, Boyd and Richerson (2002) model the conditions under which the benefits of imitating successful out-group members are sufficient to outweigh learning biases that promote individuals to learn preferentially from members of their own groups. They find that group-beneficial but individually costly traits most easily spread between groups if there is increased exposure to out-groups, and if the benefits of the trait are highly salient. High population density on the islands, considerable homogeneity across *shehia* in both social and ecological challenges to forest conservation, the use of fines by the SCC, and the prominence of the REDD + intervention on an island with relatively limited development initiatives, make each of these conditions quite feasible.

### Institutions and payoff-biased transmission

Payoff-biased transmission is further complicated when we consider the spread of institutional group-level, rather than individual-level, traits. The dynamics by which a woman imitates the tree-planting traits of a friend in her own or another community are quite different from those whereby a community adopts costly ‘institutional packages’ or sets of rules from another. The primary difference is that institutional forms are composed of sets of interlocking norms and regulations, and as such do not lend themselves to straightforward wholesale imitation (Rogers 2010). A further complication to the problem of transmitting institutional forms is that there may be a significant lag time between adoption of the new institution and its consequences, thus leading a low degree of testability (Rogers 2010). However, when there is explicit desire to learn effective institutions allows for guided variation rather than strict imitation. Guided variation, (Boyd and Richerson 1985) encourages effective SCCs to understand the fundamental causal problems involved in conservation by observing their neighbors’ institutions, efforts, challenges and successes, and thus favors them to improve existing institutional forms using causal inferences (Huang and Charman 2005).

The extent to which institutions are copied will depend on the organizational structure of the adopter. Thus, as Rogers (2010) notes, institutions are more easily adopted when decision-making (in the adopter) is centralized rather than distributed throughout the community. Thus, the of use of FPIC and democratic institutions, which is common to REDD+ and ensures project legitimacy, can reduce the rate of spread of conservation projects due to high levels of underlying heterogeneity in household economics and preferences.

The network structure that links together institutions from different groups will also affect the rate of diffusion. REDD+ projects typically have an implementing umbrella body, in the case of Zanzibar JUMIJAZA, designed specifically to coordinate the collective functioning of SCCs involved in REDD+ , and to share experiences between groups. By providing an overarching framework, JUMIJAZA creates a small world network and in turn increases the exposure of committee members to different norms and the payoffs achieved by different groups. In line with previous research (Olsson, et al. 2005), adding increased interaction between organizations dampens outgroup biases and helps committee members learn about successful institutional forms making imitation more likely. Furthermore, the umbrella NGO, in conjunction with the forestry department, are actively making decisions over where to locate scarce institutional support (particularly with respect to where to initiate new CoFMAs), and they do this expressly with respect to where they estimate the costs and benefits of REDD + adoption are most favorable, thereby further facilitating the successful spread of norms.

### Frontiers and leakage

There are additional mechanisms whereby multilevel selection can inform practitioners as to the conditions favoring the endogenous spread of conservation practices between groups. Multilevel selection predicts that when external forces create strong selection on groups, then cooperative behaviors and institutions will be more likely to emerge. In effect, external selection pressure on group resources alters the cost–benefit ratio for individuals and communities. External threats to forests, such as tree poaching, reduce a community’s immediate and long-term benefits from the forest. Adopting community-based monitoring (and REDD+ institutions) becomes more attractive—to keep neighbors out and to retain forests intact, paralleling Ostrom (2014) “demarcated boundaries” design principle.

Another common problem for REDD+ that might facilitate its spread is “leakage” (Andam et al. 2008). Leakage is used in the forestry literature to describe the protection of one’s own trees by means of harvesting those of others. REDD+ based leakage is well documented at the national and subnational scale (Nhantumbo and Camargo 2015). Individuals prevented from exploiting their own community’s forest shift their harvesting to unprotected forests in nearby neighboring communities. Communities that abut REDD+ areas are likely to be exposed to leakage, and thus they too should be more willing to adopt costly norms and institutions to protect forests from outside predation. Such dynamics bear interesting parallels to the emergence of cooperation and new political entities in zones interstitial to existing polities (Roos et al. 2015; Turchin 2003).

**Fig. 3:**
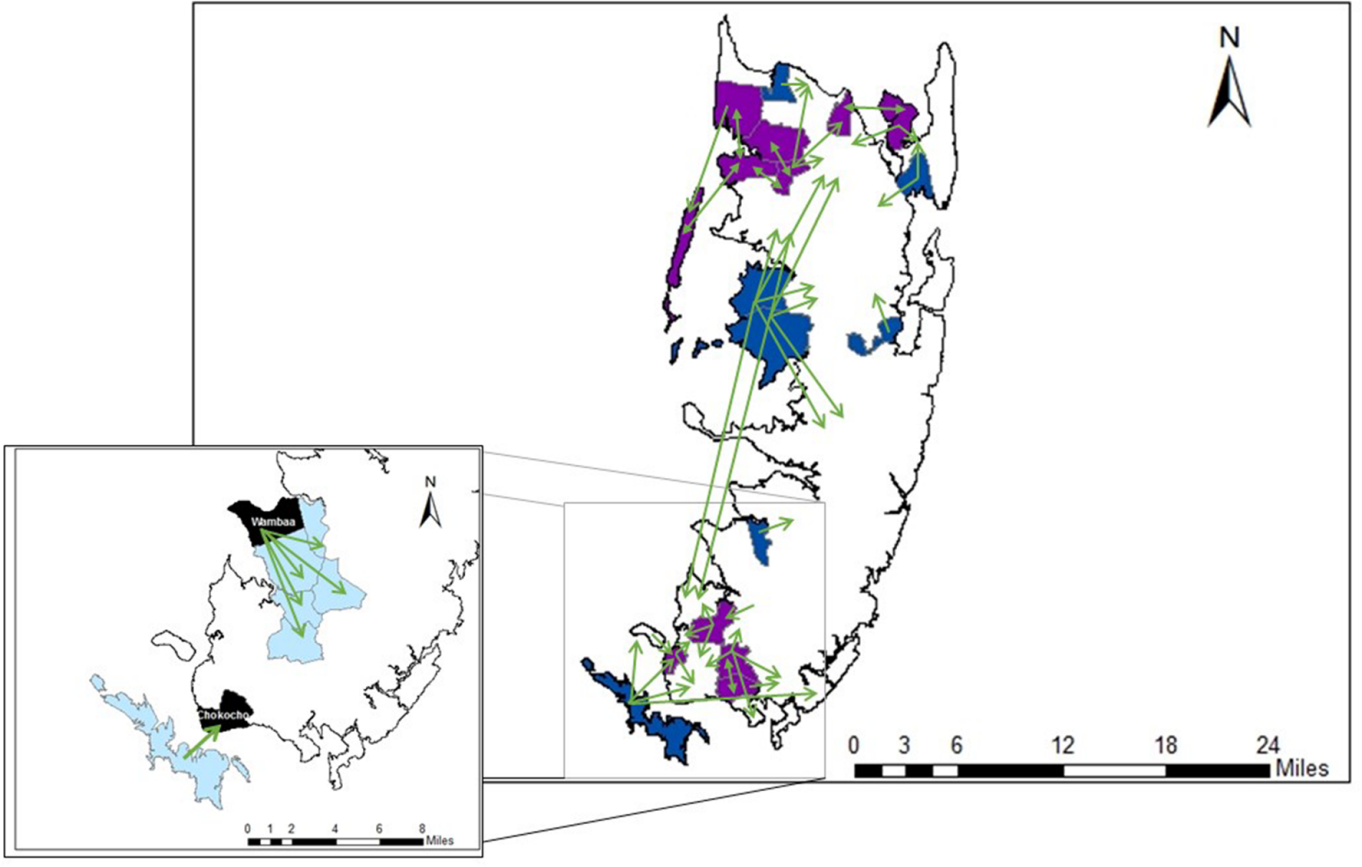
The movement of trees between *shehia* on Pemba. *Green arrows* indicate the movement of trees between *shehia*. *Blue shading* denotes *shehia* with CoFMAs that are being poached. *Purple shading* indicates *shehia* with CoFMAs that are both being poached and stealing from neighboring *shehia* (causing leakage). *Inset*. *Light blue shading* indicates existing CoFMA, *black shading* shows *shehia* with proposed CoFMAs

Poaching and leakage are prominent issues on Pemba (Fig. 3). While both are typically seen as flaws in the design of REDD+, and responsible for considerable debate over the appropriate level (national or subnational) at which REDD + projects should be implemented (O’Gorman et al. 2009), we propose that both can also promote the spread of institutional traits between neighboring groups. We anecdotally document this phenomena with the implementation of HIMA on Pemba, where two new *shehia* (Chokocho, Wambaa) are currently entering into REDD + as proposed CoFMAs (inset to Fig. 3). In the case of the proposed CoFMA at Wambaa, the goal is to protect themselves against leakage from a neighboring circle of CoFMA. At the Chokocho site the objective is to avert tree poaching on the island CoFMA lying to the south; indeed, the pressure is coming from the southern island.

We observe that more *shehia* have adopted CoFMA status on Pemba than originally planned (CARE 2014). According to forestry department personnel, this expansion results from requests either from communities suffering leakage from REDD+ compliant neighbours or from REDD+ compliant communities attempting to have better institutions instilled in their predatory neighbours. These dynamics have potential to spread institutional traits quite rapidly. In practice, the external threat acts as a group selection pressure to encourage communities to establish formal institutions to protect their forest. With relatively easily adoptable institutional packets available for imitation (CoFMAs), the REDD+ framework provides a series of unintentional, yet additional incentives to encourage forest conservation.

## Discussion

A curious reader may wonder why is it necessary to introduce yet another framework to an already crowded theoretical space (see Ostrom 1990; Gunderson 2001). Why appeal to cultural evolution when the SES framework (e.g., Folke et al. 2002) has already established a set of design principles for the sustainable management of the commons? First, as Wilson et al. (2013) argue, the design principles advocated by Ostrom (2014) (e.g., demarcated boundaries, symbolic markers, graduated punishment, and governance at nested hierarchical levels) are specific manifestations of strategic interactions that have their origin in evolutionary game theory. Second, the social complexity inherent in polycentric forms of governance is a particular example of the more general principles of cultural multilevel selection (Ostrom 2010). Third, evolutionary theory provides a synthetic, scientifically consistent account of how costly cooperative behavior emerges and spreads—this is currently absent from socio-ecological systems frameworks (Waring et al. 2015). Within the cMLS explanatory framework, sustainable institutions are no longer ahistorical manifestations of groups but instead the product of cultural evolution, and thus their origins and maintenance are open for systematic examination. By utilizing formal theory, practitioners and researchers alike are able to generate predictions as to the conditions under which sustainable, yet costly norms and institutions should endogenously develop and spread. Unlike formal theory, however, the real world is often messy. Here we will review the current challenges to implementing the REDD+ project in Pemba as a way to inform, complicate and enrich the theoretical analysis presented above.

### The distribution of REDD+ payments

For reasons discussed in Borgerhoff Mulder et al. (submitted) there have as yet been no credits sold. When they do JUMIJAZA must determine a distribution mechanism that is effective, equitable, and efficient (the ‘3Es’ Angelsen 2008; Korhonen-Kurki et al. 2014), an issue for which there are surprisingly no guide lines in the Project Document (TERRA 2017). Two questions arise: first, how to balance effort versus outcomes in assessing community success; second, how best to provide rewards. In regards to the first question, this is best decided on a project-by-project basis. In projects where communities differ in the extent to which they face outside threats to their forest, it might be most appropriate to reward effort; in projects comprising of more homogenous communities, it is best to reward according to outcome. With respect the benefit sharing mechanism, there are two primary ways whereby benefits can reach individuals. Carbon revenue can be used either to make direct payments to households, or to fund public goods supporting community socio-economic development. Both options have trade-offs. Payments made directly to individuals should either correspond to some performance-based metric or be distributed equally; either way payments should be immune to elite capture and should compensate each individual at the level of her opportunity costs (Ravikumar et al. 2017).

If revenue is used to fund public goods, the SCC faces a different set of challenges regarding how exactly the money should be spent. In Zanzibar, the Project Document (TERRA 2017) specifies that some of the REDD+ funds be directed towards alleviating the direct drivers of deforestation, through strengthening local institutions, funding planting and restoration efforts, encouraging the manufacture and sale of efficient cook stoves, and promoting economic diversification. But what proportion of funds should go to these needs, what other activities should be funded, and on whose decision? BSMs for public goods are vulnerable to elite capture and corruption, with a pessimistic estimate based on a 13 country survey suggesting as little as 10% of funds can reach community members under public goods provisioning (Pham et al. 2013). To the extent that elite capture is a problem in Zanzibar, communities must rely on JUMIJAZA to ensure financial transparency in SCCs.

### Power, institutions, inequality

Globally, REDD+ has come under increasing criticism as being a neo-liberal, neo-colonial regime that aims to commodify natural resources and restrict indigenous and community control of forests worldwide (e.g., Schroeder 2010; Fletcher and Buscher 2017). For some REDD+ represents a heinous and fundamentally flawed institution whereby hundreds of millions of the world’s poorest forest-dependent people are deceived in a manner analogous to the toxic mortgages of the 2008 housing bubble in the USA (Brown 2013). Others see REDD+ advocates as framing, validating and circulating their interventions as successes, both to maintain their coalitions of academics, consultants and NGOs, as well as to ensure financial flow (Lund et al. 2017). At the heart of these critiques is a fear that REDD+ will re-centralize forest management, thereby losing recent gains, both global (Charnley and Poe 2007) and local (Danielsen et al. 2011), with devolution over the last decade. Yet others are concerned with the challenge of attaining genuine FPIC rather than mere consultation. As in the rest of Tanzania, the REDD+ program on Zanzibar is at most sites built on a prior community forest management, thereby somewhat alleviating these concerns insofar as projects are organized at a community level and communities themselves volunteer to join the program. On the other hand, Benjaminsen (2014) describes a site on Unguja notable for elite capture, restricted FPIC and deep conflicts within the *shehia* over whether or not to persist with the program. A broader comparative study across Zanzibar (Sutta and Silayo 2014) nevertheless suggests communities are more favorably inclined towards REDD+, conforming more closely to our observations on Pemba and those in Tanzania more generally (Blomley et al. 2017) and beyond (e.g., the successes in Uganda, Jayachandran et al. 2017).

We note, however, that theory does not predict equal support among individuals within communities for participating in (or cooperating with) these institutional organizations. Instead the most efficient institutional designs are often those that recruit members of the community whom have the lowest cost–benefit ratio for inflicting punishment (Burns and Visser 2006; Flack et al. 2005). Additionally the wealthy may be best placed to benefit from the provision of public goods (Ruttan and Borgerhoff Mulder 1999). With such dynamics, one would expect the institutional organizations that support conservation, in this case the SCC, to become more politically and socially exclusive over time as elite interests infiltrate. The persistence, legitimacy and success of such institutions nevertheless depend on retaining the trust of the community, thereby providing checks and balances to power, and REDD + institutions on Pemba grant communities the power to fire and replace SCC members. Preliminary evidence suggests REDD-ready SCC in Pemba vary extensively in the trust with which they are viewed by community members, that SCC in REDD-ready *shehia* are particularly at risk to corruption allegations (Table 2), but also that effective institutions exist to replace corrupt SCC (JA field obs).

## Conclusion

Despite the general tendency for theory and practice to progress along distinct paths, the REDD + initiative opens enticing opportunities for the exchange of ideas and evidence between academia and applied science. Here we link an application of the price equation to the emergence and spread of sustainable forest management. The fundamental design principle of REDD + projects has the potential to generate a sustainable source of funding (through the sale of carbon) and evinces features compatible with the opportunity for scaling-up (through a “cooperative cascade”, Waring et al. 2015). We propose that the success of REDD+ will depend on the extent to which agents of change succeed in designing institutions that motivate the desired behavior, and at a scale appropriate for ensuring the desired outcomes. Similarly, the value of cMLS as a valuable framework will depend increasingly on the light it can shed on the patterning of empirical evidence. Despite the current challenges, both social and economic, to the REDD+ vision on Pemba (Borgerhoff Mulder et al. in prep), we anticipate value in understanding the dynamics of the spread of cooperation from a cultural evolutionary perspective. This can only enhance the effectiveness of future forest conservation institutions.
